# The Effect of Noncontingent Monetary Incentives in Recruitment Letters on Research Enrollment of Alzheimer’s Disease and Related Dementia Caregiving Dyads: A Study Within a Trial (SWAT) Randomized Controlled Trial

**DOI:** 10.21203/rs.3.rs-10818940/v1

**Published:** 2026-10-14

**Authors:** M. Yildiz, V. Winstead, A. Jacob, A. Nemariam, N. Paul, Carolyn Pickering

## Abstract

BACKGROUND Recruitment of persons living with Alzheimer’s disease and related dementias (ADRD) and their family caregivers remains challenging. Although noncontingent monetary incentives are frequently used to increase research participation, little experimental evidence supports their effectiveness for recruiting ADRD caregiving dyads. We evaluated whether noncontingent monetary incentives included in mailed recruitment letters increased study response and enrollment. METHODS This Study Within A Trial (SWAT) randomized controlled trial identified 300 potential participants through the clinical data warehouse of a US academic health system using documented dementia diagnoses and recent healthcare utilization. Participants were randomized to receive a recruitment letter with no incentive (n = 103), a $5 noncontingent gift card (n = 93), or a $25 noncontingent gift card (n = 104). Individuals who did not directly respond received follow-up telephone outreach. Outcomes included response to the recruitment letter, eligibility, and study enrollment. RESULTS Twenty-two participants (7.3%) directly responded to the recruitment letter, and 31 (10.3%) ultimately enrolled. Response rates did not differ significantly across incentive groups (χ²=1.73, p=.420). Compared with no incentive, neither the $5 incentive (OR = 1.53; 95% CI, 0.47–5.35; p=.485) nor the $25 incentive (OR = 2.06; 95% CI, 0.70–6.81; p=.204) was significantly associated with response. Age and gender were also not associated with response. Follow-up telephone outreach resulted in additional enrollments but required substantial personnel resources. CONCLUSION In this trial, $5 and $25 noncontingent monetary incentives included in mailed recruitment letters did not produce a detectable improvement in response among ADRD caregiving dyads. The low response and enrollment rates, coupled with the personnel resources required for follow-up outreach, underscore the practical challenges of recruiting caregiving dyads. Experimental evaluation of scalable, caregiver-centered, and theory-driven recruitment strategies is needed to strengthen the evidence base for efficient ADRD research recruitment.

